# Transforming Cancer Nanotechnology Data Analysis and User Experience. Part I: Current Challenges and Solutions Provided by caNanoLab

**DOI:** 10.1002/wnan.70030

**Published:** 2025-08-18

**Authors:** Weina Ke, Rui He, Mark A. Jensen, Marina A. Dobrovolskaia

**Affiliations:** ^1^ Bioinformatics and Computational Science Frederick National Laboratory for Cancer Research Sponsored by the National Cancer Institute Frederick Maryland USA; ^2^ Essential Software, Inc. Potomac Maryland USA; ^3^ Nanotechnology Characterization Laboratory, Cancer Research Technology Program Frederick National Laboratory for Cancer Research Sponsored by the National Cancer Institute Frederick Maryland USA

**Keywords:** caNanoLab, characterization, data mining, data repository, nanomedicine

## Abstract

Cancer nanotechnologies have the potential to revolutionize cancer diagnosis and treatment; however, their complexity poses challenges to data analysis and knowledge sharing. caNanoLab, a dedicated cancer nanotechnology data‐sharing portal, has emerged as a valuable resource for researchers in this field. However, to fully utilize the wealth of data available in caNanoLab, there is a need for real‐time descriptive statistical presentation and an optimized user experience. Herein, we provide an overview of cancer nanotechnologies and federally funded efforts to create data repositories, aiming to improve information flow and data sharing among researchers in the cancer nanotechnology field. We use caNanoLab as a case study to analyze the challenges in this area and highlight how caNanoLab addresses them. We also identify gaps and explore the potential of Large Language Models (LLMs) to improve user experience. A more detailed analysis of LLM and their applications to caNanoLab is provided in the second part of this review.

This article is categorized under:

Therapeutic Approaches and Drug Discovery > Emerging Technologies

## Graphical abstract

This review discusses challenges related to data complexity, management, standardization, privacy, and awareness, and how data repositories, such as caNanoLab, facilitate access to large datasets related to nanomedicine characterization. In the second part of the review, we discuss the application of Large Language Models to improve user experience with mining large datasets in public data repositories.

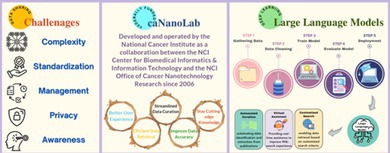

## 1 Introduction

Nanotechnology, the engineering of materials and devices at the nanoscale level, has revolutionized cancer diagnosis and treatment by allowing targeted and customized therapies with minimal side effects. Nanoparticles, with their unique physicochemical properties, biocompatibility, stability, versatility, scalability, and multifunctionality, are highly attractive subjects in cancer research, offering numerous advantages over traditional cancer diagnosis and treatments. Research in this field is ongoing, with the development of novel nanoparticle‐based approaches that may lead to more effective and customized cancer treatments. Nanoparticles are engineered to selectively target cancer cells while sparing healthy tissue, allowing for more effective and less toxic treatments. Additionally, nanoparticles are designed to release drugs selectively within tumor tissue, thereby increasing the drug's concentration at the tumor site and minimizing exposure to healthy tissue, which enhances chemotherapy's effectiveness while reducing side effects (Yan et al. 2020). For example, nanoparticles have been explored as promising tools in both photodynamic therapy (PDT) and photothermal therapy (PTT) for cancer treatment. In PDT, photosensitizing agents are activated by light of a specific wavelength, generating reactive oxygen species (ROS) that kill cancer cells. However, the clinical utility of traditional photosensitizers in PDT is limited by their poor solubility, short half‐life, and nonspecific distribution in the body. Nanotechnology platforms were explored to overcome these limitations and enhance the effectiveness of PDT. PTT utilizes light‐absorbing nanoparticles to generate heat, which can selectively target and destroy cancer cells. For example, gold nanoparticles are one of the most widely used materials in metal nanoparticles for medical applications. They have been explored as promising delivery vehicles for molecules or materials‐based PDT, either alone or in combination with chemotherapeutic agents, for cancer treatment to enhance photo‐treatment efficiency (Shang et al. 2021). PTT utilizes light‐absorbing nanoparticles to generate heat, which can selectively target and destroy cancer cells. Nanoparticles' ability to selectively accumulate in tumor tissue allows for the precise delivery of the photothermal agent to the cancerous tissue. Nanoparticles also convert light into heat more efficiently than other light‐absorbing materials, such as organic dyes or carbon nanotubes (Yang et al. 2019). Moreover, it was discovered that local application of heat‐generating nanoparticles not only destroys primary tumors but also induces a systemic anti‐tumor immune response that eradicates distant tumors (Toraya‐Brown et al. 2014). The work by Fiering et al. at Dartmouth pioneered the use of heat‐generating particles as a tool to improve immunotherapy outcomes. Likewise, the use of nanoparticles as radiosensitizers in combination with traditional radiation therapy has been found to reduce the radiation dose and improve outcomes by activating the immune response at the tumor site (Boateng and Ngwa 2019). The phenomenon of improved outcomes upon the combination of nanoparticles and traditional radio‐ or immunotherapy is known as the “abscopal effect” and has been reviewed in detail elsewhere (Morales‐Orue et al. 2019). Further development to enhance the abscopal effect involved the use of nanoparticles to capture tumor‐associated antigens (Min et al. 2017).

The immune system plays a crucial role in protecting the body against infections, diseases, and certain types of cancer. However, cancer cells can evade the immune system by producing proteins that suppress immune responses or by altering their surface molecules to avoid detection by immune cells. Nanoparticles have demonstrated significant potential in enhancing the efficacy of immunotherapy in cancer treatment. In addition to the use of nanoparticles for improving the abscopal effect, another use of nanotechnology in cancer immunotherapy included the combination of immune checkpoint inhibitors and immunogenic cell death (ICD) inducers and using nanoparticles to co‐deliver them to reverse immunosuppression, prevent tumor metastasis and recurrence, and improve the efficacy of cancer treatment [6]. Other efforts at using nanoparticles to improve the outcome of immunotherapy with nanotechnology have involved metabolic reprogramming of the tumor microenvironment, increasing tumor infiltration with lymphocytes, decreasing the number of M2 macrophages and other immunosuppressive cells, and increasing the overall immunogenicity of the tumor (Kim et al. 2021; Ren et al. 2022; Som et al. 2019; Zang et al. 2022).

Gene therapy involves the introduction of genetic material into cells to treat or prevent diseases, such as cancer or genetic disorders. However, the delivery of genetic material to target cells can be challenging, as it must overcome several biological barriers, including enzymatic degradation, rapid clearance from the circulation, and uptake by non‐target cells. Nanoparticles can overcome these challenges by protecting the genetic material from degradation, increasing its stability, and enabling targeted delivery to the desired cells. Different types of nanoparticles, such as liposomes, polymeric nanoparticles, and viral vectors, have been developed for gene delivery (Mirón‐Barroso et al. 2021). Moreover, a sophisticated class of nucleic acid‐based nanoparticles—DNA origami, RNA nanoparticles, DNA and RNA/DNA hybrid nanoparticles—also known as Nucleic Acid Nanoparticles (NANPs) have been developed and tested for gene delivery and immunomodulation with success (Afonin et al. 2022; Chandler et al. 2022; Du et al. 2022; Krishnan and Bathe 2012; Li et al. 2022; Li et al. 2023; Newton et al. 2023; Veneziano et al. 2020; Wang et al. 2021; Wang and Guo 2021; Zhovmer et al. 2021). The technology used to create DNA origami involves a long single‐stranded DNA scaffold that is folded into desired shapes using short DNA oligonucleotides, known as “staples.” In contrast, RNA, DNA, and RNA/DNA nanoparticles are assembled from shorter RNA, DNA, or DNA/RNA hybrid oligonucleotides (Afonin et al. 2010; Afonin et al. 2011; Afonin et al. 2014; Krishnan et al. 2012; Veneziano et al. 2020). Furthermore, advances in gene editing technologies, such as CRISPR/Cas9, have enabled the development of nanoparticles that can deliver gene editing tools directly to cancer cells to target specific genetic mutations (Duan et al. 2021).

Monoclonal antibodies are laboratory‐produced molecules designed to target specific cancer cells and minimize damage to healthy cells. However, the clinical efficacy of monoclonal antibodies can be limited by their poor pharmacokinetics, poor bioavailability, and low tumor penetration. Nanoparticles not only overcome these limitations but also enhance their functionality by enabling the co‐delivery of multiple therapeutic agents (Wathoni et al. 2022). Cancer vaccines stimulate the immune system to recognize and attack cancer cells, whereas their efficacy is limited by poor antigen presentation on tumor cells. Targeting human dendritic cells via DEC‐205 using PLGA nanoparticles leads to enhanced cross‐presentation of a melanoma‐associated antigen (Saluja et al. 2014). The poor immunogenicity of live tumor cells is another obstacle, likely due to the secretion of soluble factors that can suppress immune cells (Chiang et al. 2010). Therefore, various approaches are often employed to enhance the immunogenicity of tumor cells and enhance the efficacy of whole‐tumor cell vaccines. The interferon genes (STING)‐activating nanoparticles exhibit strong immunogenicity, and adjuvants such as CpG motifs, polymers, liposomes, and small molecule agonists are also often used to enhance the immunogenicity of vaccines (Liu et al. 2022).

In terms of diagnostic imaging, nanoparticles have been utilized as contrast agents to enhance the accuracy and specificity of cancer detection. For example, iron oxide nanoparticles are used in magnetic resonance imaging (MRI) to enhance the contrast between tumor tissue and normal tissue (Ahmadpoor et al. 2021). In another aspect of diagnosis, nanosensors can detect biomarkers associated with cancer at low concentrations, enabling earlier and more accurate diagnosis. These nanosensors can also be used to monitor the effectiveness of cancer treatments in real time, enabling customized and precise therapy (Swierczewska et al. 2012).

Nanotechnologies also apply to combination therapy, which involves the use of two or more treatments, such as chemotherapy, radiation therapy, immunotherapy, or targeted therapy, together to enhance their effectiveness and reduce the risk of resistance. This approach has become increasingly popular in cancer treatment, as it can improve patient outcomes and potentially reduce treatment‐related toxicity. One example is the use of nanoparticles to co‐deliver chemotherapy drugs and immunomodulatory agents. This approach has been shown to enhance the anti‐tumor immune response while also reducing the toxicity associated with chemotherapy. For example, researchers have developed a nanoparticle‐based therapy that co‐delivers the chemotherapy drug doxorubicin and the immune checkpoint inhibitor NLG919 (Sun et al. 2017). The nanoparticles are designed to release the drugs in a controlled manner at the tumor site, leading to enhanced tumor regression and prolonged survival in a pre‐clinical model of breast cancer. Another example is the use of nanoparticles to deliver both therapeutic agents and diagnostic agents. This approach, known as theranostics, enables the real‐time monitoring of therapeutic responses, leading to improved treatment outcomes. For example, a multifunctional theranostic nanoparticle was fabricated to enhance tumor‐targeted imaging and promote focused ultrasound (FUS) ablation, chemotherapy, and sonodynamic therapy (SDT) (Kang et al. 2022).

The field of cancer nanotechnology has been making remarkable progress, with the development of new and improved nanomaterials that can target cancer cells more specifically, deliver therapeutic agents with greater efficacy, and minimize side effects. However, the challenges in data analysis, sharing, and management have become increasingly apparent due to the sheer volume of data generated. Overcoming these challenges will be a crucial goal in advancing research and discovery in cancer nanotechnology, as well as accelerating the development of safe and effective nanotherapies for cancer. The breadth of nanotechnology carriers, active ingredients, and applications, along with the extensive studies conducted over the past two decades, has generated a substantial volume of data that can be leveraged to achieve this goal. Herein, we discuss one such data platform—caNanoLab.

## 2 Challenges in Cancer Nanotechnology Data Analysis, Sharing, and Management

Nanotechnology is rapidly expanding its presence in the field of cancer research and treatment. One of the primary focuses is on the development of new and improved nanomaterials that can target cancer cells with higher specificity and deliver therapeutic agents with greater efficacy while minimizing side effects. A key component of this work is the investigation of the biological interactions between nanomaterials and living organisms, including their potential toxicity and immunogenicity (Kemp and Kwon 2021). As the field continues to grow, researchers are generating a wealth of data on nanomaterials synthesis, physicochemical properties, and in vitro and in vivo characterizations, activities, and functions (Ke et al. 2022). This valuable data is scattered across diverse research fields and publications. The need to identify and gather relevant cancer nanotechnology data, validate its quality, store and share it effectively, and apply it for analysis and reporting is becoming increasingly important.

Although we are still in the early stages of development, we believe it is imperative to address the following challenges in data analysis, data sharing, and management to accelerate progress in cancer nanotechnology.

### 2.1 Challenge 1: The Complexity of the Data

The complexity of cancer nanotechnology data presents a significant challenge to researchers when attempting to extract relevant information from the vast amount of available data. The data generated from experiments conducted using nanomaterials can be highly diverse and complicated, making it challenging to analyze and interpret. The sheer volume of data available in the literature can be overwhelming, and researchers may struggle to identify the specific data they need for their analysis. This issue is compounded by the fact that the data generated is often unstructured, meaning it is not arranged in a specific format or order, making it difficult to organize and analyze. Moreover, the variability in data quality and consistency poses another significant challenge for researchers. Data collected from experiments can vary in quality and consistency due to several factors, including experimental conditions, the accuracy of measurement instruments, and the experimenter's skill. This variability can lead to errors and inaccuracies in data analysis, making it challenging to draw meaningful conclusions from the results.

### 2.2 Challenge 2: Lack of Standardization

The lack of standardization in data collection and reporting across different research fields and databases is another significant challenge in data analysis, sharing, and management. Currently, there are no standardized protocols for characterizing nanomaterials, and different laboratories may employ varying methods to measure the same properties. This variability in data collection and reporting can lead to inconsistencies in the data, making it difficult to compare and combine data from different sources. Additionally, there is a lack of standardized vocabularies and ontologies for describing nanomaterial properties and biological interactions, which leads to ambiguity and confusion in data interpretation and hinders cross‐database comparison and integration. Moreover, the lack of standardization can also affect the reproducibility of experiments and the reliability of the data. Without clear and consistent reporting standards, it is difficult to replicate experiments and verify results; this can lead to potential discrepancies in the data. Furthermore, the lack of standardization in data formats hinders seamless data sharing across various platforms and databases. Researchers and scientists may use different file formats, data models, and data dictionaries, which makes it challenging to integrate and analyze the data effectively, resulting in significant barriers to data sharing and accessibility and limiting the potential for collaborations and discovery.

### 2.3 Challenge 3: Poor Data Management Practices, Incomplete or Inaccurate Metadata, and Low Data Quality

Poor data management practices can lead to severe consequences, including lost, corrupted, or duplicated data, which makes it difficult for researchers to locate and access the necessary data. Without proper storage, labeling, and documentation, it can be difficult for researchers and their collaborators to track changes to the data over time or to ensure it is properly validated and verified. Data that is not adequately protected or managed is vulnerable to data breaches or misuse, potentially exposing sensitive patient information and compromising the integrity of research.

Metadata provides important context about the data, helping researchers understand how to use and interpret it effectively. Incomplete or inaccurate metadata can lead to misunderstandings and errors in analysis, affecting the quality and reliability of the data and making it difficult for researchers to use the data effectively. Metadata can include information such as the date of data collection, source, and methodology used to collect the data. Incomplete or inaccurate metadata can lead to misunderstandings and errors in analysis, affecting the quality and reliability of the data and making it difficult for researchers to use the data effectively, which can result in misinterpretation or incorrect conclusions. Inaccurate or incomplete metadata also impacts data reuse. Haphazardly described data presents obstacles for other researchers to understand and utilize the data for their own research purposes. Finally, low‐quality data may contain errors, inconsistencies, or bias, not only making it difficult for researchers to draw meaningful conclusions or identify important trends and patterns in the data, but also leading to the misinterpretation of research findings, which can potentially result in ineffective therapies and negatively impact patient care. This underscores the importance of high‐quality data in cancer nanotechnology research and the need for rigorous quality control measures to ensure that data is accurate and reliable.

### 2.4 Challenge 4: Data Privacy and Confidentiality

Data privacy and confidentiality are significant concerns that discourage researchers from sharing their data. Firstly, cancer nanotechnology research frequently involves sensitive and confidential data, such as personal health information, which must be protected to prevent unauthorized access or misuse. Researchers may hesitate to share such information due to the potential ethical and legal implications of mishandling or misusing the data, such as loss of patient trust, regulatory penalties, and legal liabilities. Secondly, the interpretation and analysis of cancer nanotechnology data can be complex and challenging, leading to incorrect conclusions or harm to patients if not properly handled. Researchers may be concerned about the quality and accuracy of the data and may hesitate to share it until they are confident that it has been adequately analyzed and interpreted. Thirdly, there may be concerns about academic credit and recognition. Researchers invest significant amounts of time and resources in collecting and analyzing data, and sharing this data makes it challenging for them to maintain control over their research findings. Researchers may also worry that sharing data could lead to the publication of additional research papers by other researchers, who may not acknowledge or credit the original researchers. Fourthly, legal and ethical issues may hinder data sharing, including regulatory compliance, patient privacy, intellectual property, and obtaining informed consent. Researchers must adhere to strict regulatory and legal requirements to ensure that patient privacy is protected and informed consent is obtained before sharing any data. Failure to comply with these regulations can result in severe consequences, including legal and financial penalties, damage to reputation, and loss of research funding. Finally, there may be cultural or institutional barriers to the sharing of data. For example, researchers may be accustomed to working in a competitive research environment, where sharing data could be seen as giving an advantage to competitors. Institutions may also lack clear policies and guidelines around data sharing, making it difficult for researchers to know how and when to share their data. These concerns make it challenging for researchers to share their data, even when doing so could accelerate the development of safe and effective nanotherapies for cancer.

### 2.5 Challenge 5: Lack of Public Awareness and Funding

Developing and maintaining databases requires significant investments in terms of time, personnel, and infrastructure. This includes developing and implementing data collection and management systems, establishing quality control measures, and ensuring the security and privacy of data. Additionally, ongoing maintenance, updates, and improvements are essential to ensure the database remains relevant and valuable to the scientific community. The importance of cancer nanotechnology databases and their potential impact on cancer diagnosis and treatment may not be fully appreciated by some individuals and organizations; this can result in inconsistent and insufficient funding support for database development and maintenance. Private donors may be inclined to support research in more widely recognized areas, leading to a lack of resources for public databases. On the other hand, many databases are currently maintained by academic institutions or non‐profit organizations, which rely on external funding sources, such as government or industrial grants. These funding sources are often limited and competitive, making it difficult for smaller organizations to sustain long‐term operations. As a result, many databases are at risk of being shut down or not being updated regularly, leading to data gaps and inconsistencies. Another fact is that funding for nanotechnology research is also limited, and competition for resources can be intense, making it challenging for public databases to secure the necessary resources to establish and maintain high‐quality data management systems. Therefore, some public databases for nanotech data mining may struggle to remain up‐to‐date and fully functional, which can ultimately limit their usefulness for cancer nanotechnology research.

## 3 National Cancer Institute Alliance for Nanotechnology in Cancer

NCI recognized the value of nanotechnology in medicine early. It funded a large and comprehensive program called the Alliance for Nanotechnology in Cancer in 2004. At its initial iteration, the program consisted of large multi‐disciplinary Centers of Cancer Nanotechnology Excellence (CCNEs), smaller, single‐investigator research projects, and training programs to develop a multi‐disciplinary workforce (Hartshorn et al. 2019). CCNEs operated for 15 years (Grodzinski 2019) and produced not only a large body of scientific knowledge but also enabled the formation of several start‐up companies focused on commercializing technologies developed in academia. Currently, the NCI continues to fund cancer nanotechnologies through R01 grants—the most common NIH funding mechanism. These R01 grants are dedicated to ongoing mechanistic studies aimed at furthering our understanding of nanoparticle and nano‐device interactions with biological systems, thereby enabling the design of more effective cancer nanotechnology interventions. More mature nanotechnology concepts are supported for the continued development of pre‐clinical data to enable future translation. Two resources established by the NCI Alliance for Nanotechnology in Cancer, the Nanotechnology Characterization Laboratory and caNanoLab, have been reviewed in detail earlier (Ke et al. 2022). Herein, we focus on the data repository resource, caNanoLab, and use it as a case study to analyze challenges in data sharing, current advances in improving the user interface, and pave the way for future efforts in the field.

## 4 caNanoLab, a Specialized Data‐Sharing Platform for Cancer Nanotechnology

**FIGURE 1:**
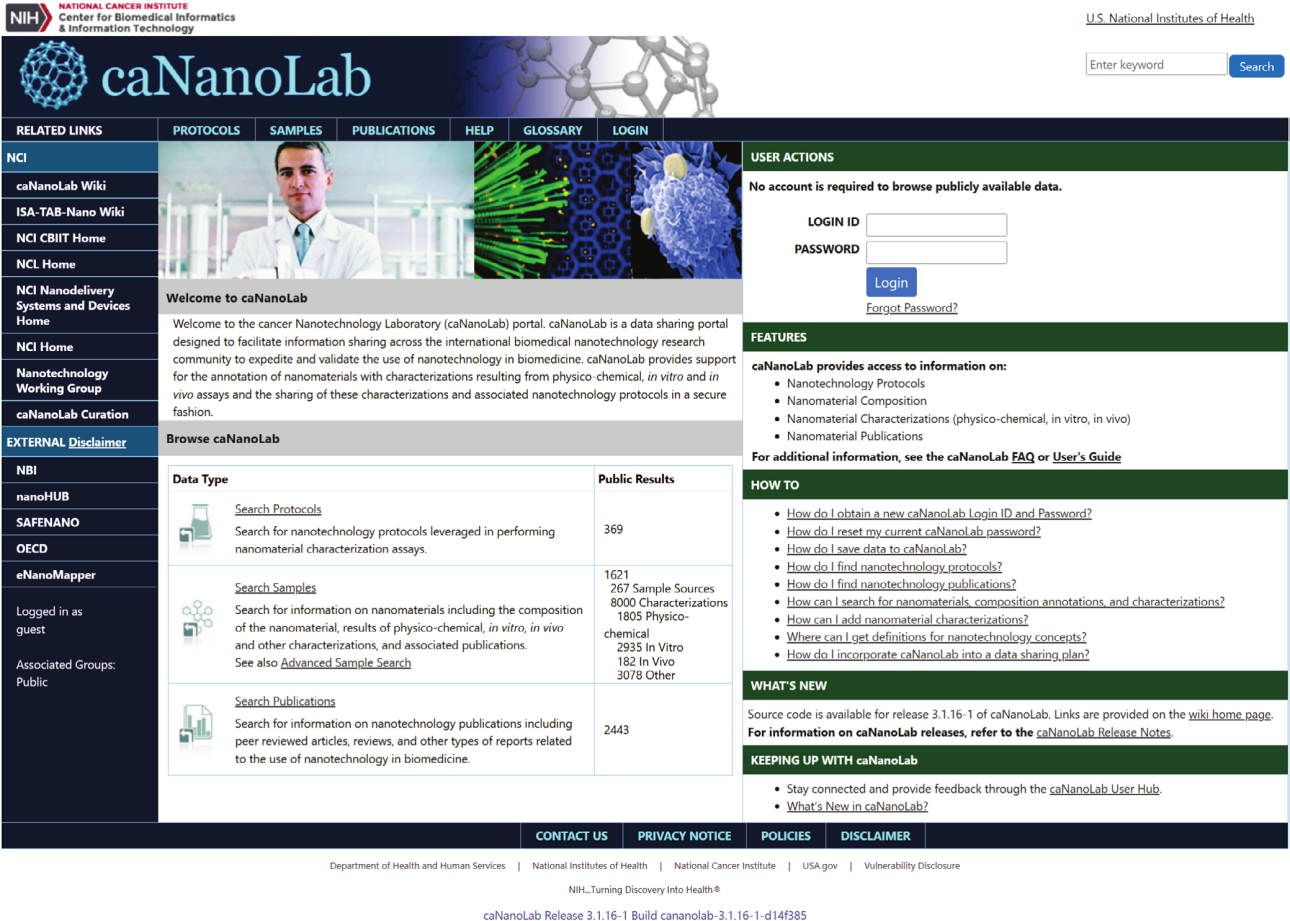
caNanoLab home page. The image displays the homepage of the caNanoLab data repository https://cananolab.cancer.gov/#/ intended for data sharing and mining to improve development of cancer nanomedicines.

The cancer Nanotechnology Laboratory (caNanoLab) (https://cananolab.cancer.gov/) is a dedicated data repository and data sharing portal for cancer‐related nanotechnology research, providing the international biomedical nanotechnology research community with the ability to expedite and validate the use of nanotechnology in biomedicine (Figure 1). Since its inception 17 years ago, caNanoLab has been providing the biomedical research community with reliable and up‐to‐date information on cutting‐edge nanotechnology in cancer diagnosis and treatment. The platform offers several advantages to researchers and scientists in the field of cancer nanotechnology, including a comprehensive database that houses a wealth of information on nanomaterials, protocols, and biological interactions, as well as a highly customizable interface that enables the creation and sharing of custom submissions, facilitating collaboration with other researchers.

The specialized focus of caNanoLab on cancer‐related nanotechnology data is a key factor that sets it apart from other general nano‐databases. While other databases may encompass a range of nanotechnology research, caNanoLab's exclusive focus on cancer‐related nanotechnology enables a more targeted and specialized approach to research in this field. This specificity of caNanoLab also allows researchers to conduct more in‐depth and specialized research into cancer nanotechnologies. By focusing exclusively on cancer‐related nanotechnology data, researchers can access more specialized and specific information on the application of nanotechnology in cancer diagnosis and treatment. This highly detailed and nuanced data allows researchers to develop a more comprehensive understanding of the complexities of cancer and how nanotechnology can be used to combat it. The specialized focus also encourages collaboration among researchers in the same field, facilitating the exchange of knowledge and ideas and advancing research in the field of cancer nanotechnology. Furthermore, this specialized focus can potentially accelerate the design and optimization of next‐generation nanomedicine. By accessing highly specific and relevant information about nanomaterials, their composition, function, physicochemical, in vitro, in vivo, and ex vivo characterizations, and biological interactions, researchers can make more informed decisions about the use of cancer nanotechnology. This, in turn, can lead to the development of more effective and efficient nanomedicine, resulting in better patient outcomes.

**FIGURE 2:**
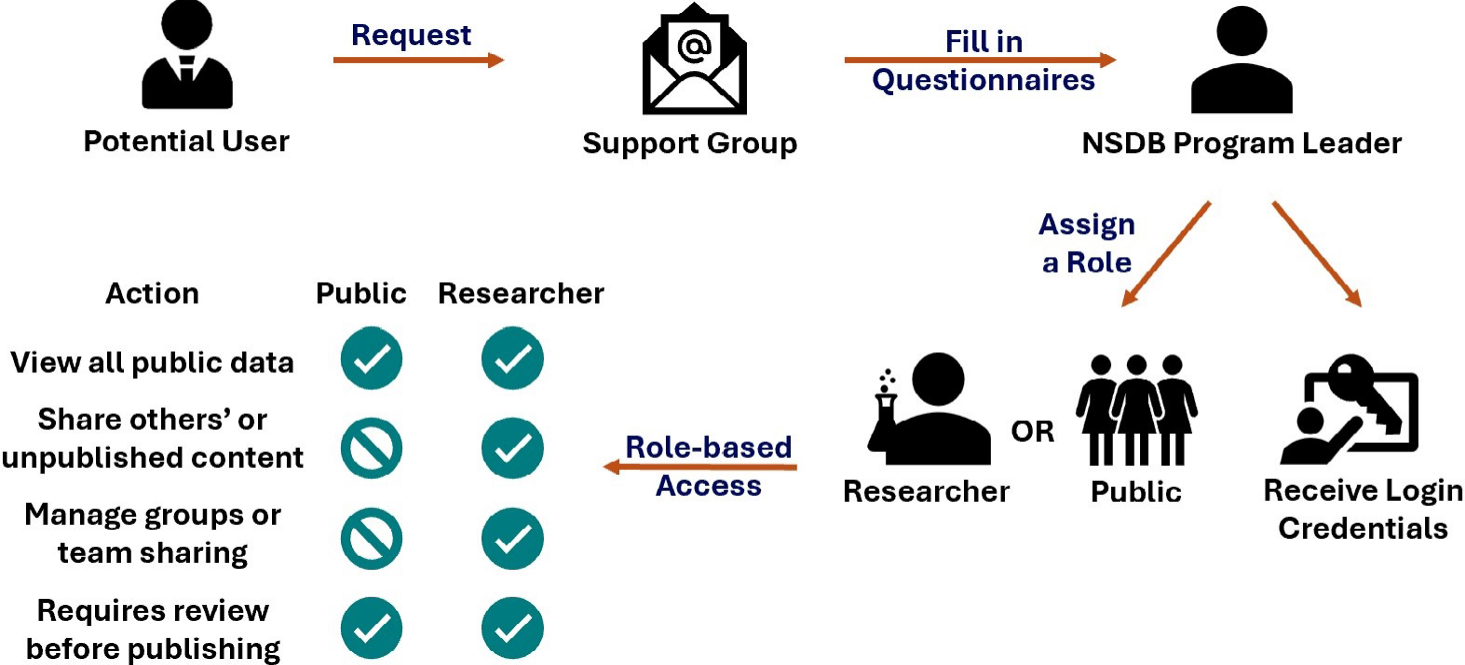
caNanoLab user registration workflow. This diagram illustrates the user registration workflow for caNanoLab, as well as the role‐based access permissions assigned to the Public and Researcher roles. More details about the actions that can be performed by the Researcher and Public roles are provided on the caNanoLab wiki, which can be accessed at https://wiki.nci.nih.gov/spaces/caNanoLab/pages/339381149/caNanoLab+User+Roles. NSDB—Nanodelivery Systems and Devices Branch https://dctd.cancer.gov/programs/cip/about/nano.

caNanoLab is open to any extramural and NIH intramural investigators interested in cancer nanotechnology. To register for a caNanoLab account, a user must submit a registration request to the caNanoLab support team via email. Support staff reply with a short questionnaire that captures the applicant's institutional affiliation, job title, research focus, intended use of caNanoLab, and anticipated data‐sharing needs. When the completed questionnaire is returned, the support group forwards it to the Nanodelivery Systems and Devices Branch (NSDB) program leader, who verifies the information and assigns the appropriate account type within several business days. Approved applicants receive either a public role, which allows viewing all public records and uploading data for the curator to review, or a researcher role, which allows full access to shared/non‐public data, the ability to create collaboration groups, and broader content‐sharing privileges. Once the role is set, the user receives login credentials and a link to establish a password, after which they can begin working in caNanoLab (Figure 2).

**FIGURE 3:**
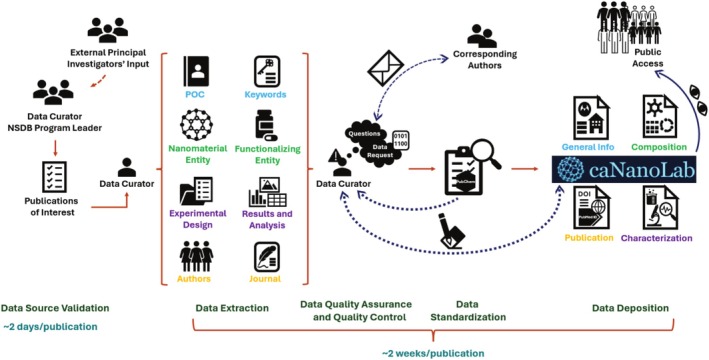
caNanoLab in‐house data curation workflow. This diagram outlines the internal workflow used by data curators to extract, validate, standardize, and deposit nanomaterial details and characterization data into caNanoLab.

**FIGURE 4:**
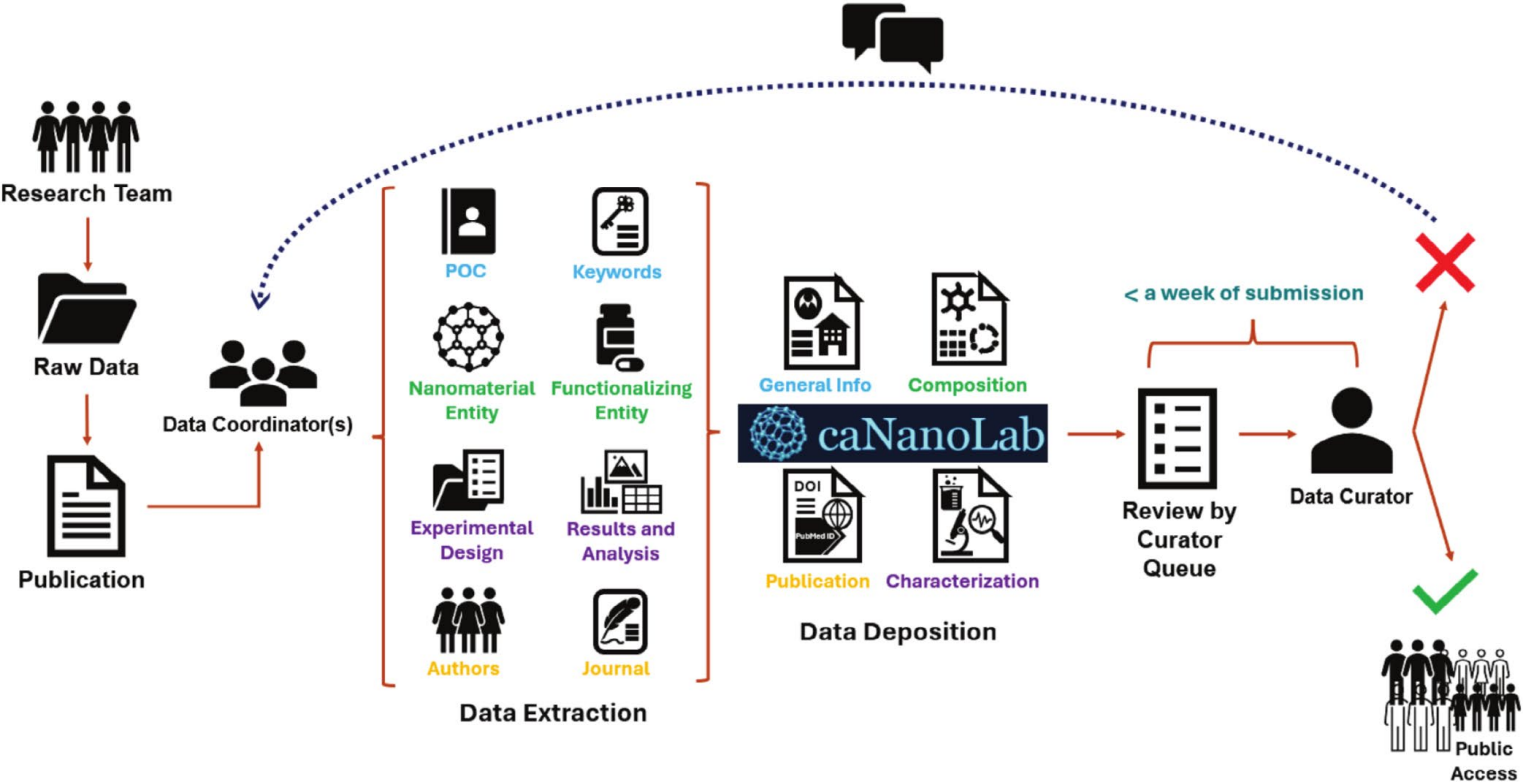
caNanoLab Data Coordination and Curation Workflow. This diagram illustrates the data submission and review process led by data coordinators, with final approval and public release managed by the caNanoLab curator.

Schematics that map out the complete data submission and processing workflow, annotated with estimated duration for each stage, are presented in Figures 3 and 4. The procedures for in‐house data curation, including publication selection, validation, quality control, and data deposition, are illustrated in Figure 3. This curator‐driven process begins with the identification of publications of interest by the data curator and NSDB program leader, sometimes incorporating input from external principal investigators. The curator extracts key data elements, including nanomaterial entities, functionalizing components, experimental design, and results. When information is unclear or missing, curators contact the corresponding authors to request clarification or additional data. All extracted content undergoes quality assurance and standardization steps, including verification against trusted resources such as PubChem. After a thorough review, the curated data is structured into specific sections within caNanoLab—such as general information, composition, characterization, and publication metadata—and becomes publicly available. This rigorous in‐house workflow ensures consistency, accuracy, and usability across records, with a typical curation timeline of approximately 2 weeks per publication.

The data coordination and curation workflow from the perspective of data coordinators is illustrated in Figure 4. First, research teams generate raw data and publish their findings. Next, data coordinators, often members of the research team, organize and submit the extracted data, including nanomaterial characterizations, protocols, and publication details, into caNanoLab. Once submitted to the curator, the data enters the curator's review queue. The data curator routinely checks the curation queue and begins processing submitted entries within 1 week of user submission. The data curator ensures completeness, accuracy, and adherence to curation standards. If the submission meets quality criteria, it is approved and made publicly accessible via caNanoLab. If additional clarification or data is needed, the curator engages with the data coordinators to resolve outstanding issues prior to release.

caNanoLab provides a feasible workflow for researchers to submit and retrieve information on nanoparticles, including their composition, function, physicochemical properties, in vitro, in vivo, and ex vivo experimental characterizations. caNanoLab's distinctive customized visibility feature empowers submitters to control who can access their data. This feature provides submitters with options at three levels of data visibility: private, collaboration group, or public access, allowing them to tailor their submission to their specific needs and preferences. With this level of control, researchers can ensure that their data is shared with the appropriate audience while maintaining confidentiality and privacy when necessary. Once the data is submitted, it is reviewed by the curator to ensure that it meets the established standards and quality control measures. The curator checks for any discrepancies, errors, or inconsistencies in the data and works with the submitter to resolve any issues that may arise. This review process is crucial in ensuring that the data in the caNanoLab database is accurate, consistent, and of high quality. After the data has been curated and approved, it becomes publicly accessible for search and analysis by researchers and scientists worldwide.

Collaboration, data sharing, and comparison across different studies are facilitated, promoting the dissemination of knowledge within the nanotechnology community.

Researchers can utilize the customizable search function to stay current on the latest advancements in cancer nanotechnology. In addition to providing searchable data and protocols for nanomaterial characterization and synthesis, the platform also features cutting‐edge publications related to nanomedicine research. The search function is highly customizable, allowing researchers to tailor their search to include specific parameters, such as nanoparticle size, shape, and surface charge. Furthermore, researchers can refine their search by various criteria, such as biological assay results. This level of customization helps researchers find relevant information quickly and efficiently. The output of the search results can be exported in either XML or JSON format, providing flexibility and ease of use for researchers who need to integrate the results into their own systems. This feature allows researchers to use the information they find in caNanoLab in their own studies and analyses.

In addition to the customizable search function and data submission options, caNanoLab also offers several tools and features that facilitate collaboration among researchers.

One of the key features is the ability for registered users to create collaboration groups. These groups enable researchers working on similar projects to share data and collaborate with one another in a secure and controlled manner. By creating a collaboration group, researchers can easily invite others to view and contribute to their data, as well as receive feedback and insights from their peers. Collaboration groups can be especially beneficial for larger research projects that involve multiple teams or institutions. With caNanoLab, researchers from various locations can easily share their data with one another, thereby reducing the need for time‐consuming and expensive data transfers. Additionally, collaboration groups help ensure that all team members are working with the most up‐to‐date data, as changes made by one team member can be instantly shared with the rest of the group. This collaboration may also lead to new research opportunities, as researchers may identify potential areas for collaboration or pinpoint areas where further investigation is necessary.

caNanoLab has actively collaborated with several organizations to establish standards for nanotechnology data. The Nanotechnology Information Object Model (nano‐OM) is a standardized framework for organizing and sharing nanotechnology data, serving as the foundation for caNanoLab's data architecture. nano‐OM supports the capture of parameters recommended by the Minimum Physical and Chemical Parameters for Characterizing Nanomaterials on the Toxicology Initiative (Gaheen et al. 2013). caNanoLab utilizes the nano‐OM to capture and represent data on nanomaterials submitted to the database, ensuring consistency and standardization of data across different studies. The use of a standardized data model also facilitates the interoperability and integration of different databases and information systems, enabling the seamless exchange and comparison of nanotechnology data. The Nanoparticle Ontology (NPO) is a standardized vocabulary and classification system used by caNanoLab to define nanomaterial samples based on their composition, characterization, and associated publications (Olsen 2022). NPO defines concepts for nanoparticle entities, assays, and protocols, and enables the organization of data based on specific categories, facilitating data sharing and comparison across different studies. NPO also provides a way to ensure consistency and accuracy in data collection and analysis, as well as enabling the integration and interoperability of different databases and information systems (Thomas et al. 2011).

To ensure a unified and standardized approach towards data reporting and sharing, caNanoLab has adopted the Minimum Information about Nanomaterials (MIN) guidelines into its data curation and reporting standards. This set of guidelines, developed by a consortium of esteemed experts from academia, industry, and government agencies in 2011, covers an extensive range of topics pertaining to nanomaterial characterization (Faria et al. 2018). The objective of these guidelines is to enhance the consistency and quality of nanomaterial data reporting, thereby facilitating the seamless sharing and integration of data across multiple platforms and databases. By implementing these guidelines during the evaluation of publications for curation and submissions from researchers, caNanoLab has raised the bar for the quality and reliability of nanotechnology research data.

caNanoLab has undergone numerous upgrades since its inception, with more than 40 upgrades released to provide improved usability features from version 0.5 to the latest version 3.1.4. These upgrades have brought a host of sophisticated usability features to the forefront, including enhanced support for characterization metadata, streamlined end‐user data entry and annotation, robust search functions, an expanded limit for CSV and publication uploads, superior protocol versioning management, cross‐referencing to PubMed publications, and refined printing and export functions. Additionally, security has been enhanced, and the platform has migrated to a cloud‐based architecture, resulting in significantly improved flexibility and scalability. These enhancements demonstrate NCI's ongoing commitment to standardization and the development of high‐quality datasets, as well as the creation of data‐sharing and analytical tools that significantly enhance the value of cancer‐focused biomedical research.

caNanoLab has made significant contributions to the field of cancer nanotechnology research since its inception 17 years ago. The platform has also received significant contributions in the form of over 1500 nanomaterial data submissions, more than 2000 publications, and approximately 200 protocols. These numbers are a testament to the active engagement and valuable contributions of the research community within caNanoLab. As a widely used and highly respected platform in the biomedical research community, caNanoLab stores extensive data on the synthesis, properties, and characterization of nanomaterials. Within this vast repository of information lie important trends and insights that shed light on new directions in the field of nanotechnology (Ke et al. 2022). caNanoLab has been cited in over 200 peer‐reviewed publications and is an open‐source, freely accessible resource to users worldwide. The impact of caNanoLab on the field of cancer nanotechnology is evidenced by its recognition as a critical resource by several prominent organizations, including the National Cancer Institute, Alliance for Nanotechnology in Cancer, and the Nanotechnology Characterization Laboratory (Morris et al. 2014).

caNanoLab is the only federally funded, outward‐facing data repository dedicated to cancer nanotechnology data. The voluntary nature of data deposition by cancer nanotechnology researchers may limit the amount of data available through caNanoLab when compared to the number of published papers in the field. To overcome this limitation and ensure representation of various nanotechnology platforms in caNanoLab, the curator identifies and enters fundamental papers on nanotechnologies not currently represented in the database. The trends in cancer nanotechnology types and their uses for various cancer indications represented in caNanoLab match those seen by another federally funded resource—the National Cancer Institute's Nanotechnology Characterization Laboratory—and the US Food and Drug Administration, as was reviewed in detail elsewhere (Crist et al. 2025; Ke et al. 2022). With ongoing upgrades and enhancements to its features and functionality, caNanoLab remains at the forefront of cancer nanotechnology research and is poised to continue making significant contributions to the field in the years to come.

## 5 Conclusion

caNanoLab has indisputably risen to prominence as a premier platform for managing, sharing, and analyzing data from cancer nanotechnology research. The platform's remarkable success in providing researchers with a structured and comprehensive database of nanomaterials and their properties, as well as characterization protocols and associated publications, has garnered widespread acclaim. However, this achievement is primarily attributed to the dedicated efforts of a team of professionals, whose commitment has been instrumental in ensuring the quality, accuracy, and integrity of its multifarious data. Nevertheless, the diverse and intricate nature of caNanoLab's data can be daunting for newcomers, and the frequent implementation of novel features and updates requires a certain level of training to navigate the portal effectively. With the advent of Large Language Models (LLMs), caNanoLab now has the potential to leverage automation and artificial intelligence to enhance its usability, which we believe will reduce the burden on human resources for training new users and addressing system‐related queries. In the second part of this review, we will delve into this advancement in greater detail.

## Author Contributions

**Weina Ke:** conceptualization (equal), data curation (equal), formal analysis (equal), visualization (equal), writing – original draft (equal), writing – review and editing (equal). **Rui He:** conceptualization (equal), data curation (equal), formal analysis (equal), visualization (equal), writing – original draft (equal), writing – review and editing (equal). **Mark A. Jensen:** resources (equal), supervision (equal), visualization (equal), writing – original draft (equal), writing – review and editing (equal). **Marina A. Dobrovolskaia:** project administration (equal), resources (equal), supervision (equal), writing – original draft (equal), writing – review and editing (equal).

## Conflicts of Interest

The authors declare no conflicts of interest.

## Data Availability

Data sharing does not apply to this article as no new data were created or analyzed in this study.
